# Two-Year Follow-Up Analysis of Telaprevir-Based Antiviral Triple Therapy for HCV Recurrence in Genotype 1 Infected Liver Graft Recipients as a First Step towards Modern HCV Therapy

**DOI:** 10.1155/2016/8325467

**Published:** 2016-04-18

**Authors:** Fritz Klein, Ruth Neuhaus, Dennis Eurich, Jörg Hofmann, Sandra Bayraktar, Johann Pratschke, Marcus Bahra

**Affiliations:** ^1^Department of General, Visceral and Transplantation Surgery, Charité Universitätsmedizin Berlin, Campus Virchow, Augustenburger Platz 1, 13353 Berlin, Germany; ^2^Institute of Medical Virology, Helmut-Ruska-Haus and Labor Berlin Charité-Vivantes GmbH, Charité Universitätsmedizin Berlin, Augustenburger Platz 1, 13353 Berlin, Germany

## Abstract

*Objective.* The introduction of protease inhibitors telaprevir and boceprevir in 2011 had extended the antiviral treatment options especially in genotype 1 infected hepatitis C relapsers and nonresponders to interferon/ribavirin therapy. The aim of this study was to analyze the long-term treatment efficiency of telaprevir-based triple therapy for patients with hepatitis C reinfection after orthotopic liver transplantation.* Patients and Methods.* We included 12 patients with histologically confirmed graft fibrosis due to hepatitis C reinfection. The treatment duration was scheduled as 12 weeks of telaprevir-based antiviral triple therapy followed by 36 weeks of dual therapy with pegylated interferon/ribavirin. The patients were followed up for two years after the end of triple therapy.* Results.* Of the 12 patients, 6 (50%) completed the full 48 weeks of antiviral treatment. An end of treatment response and a sustained virological response 52 weeks after the end of the antiviral treatment course were achieved in 8/12 (67%) and 7/12 (58%) patients, respectively.* Conclusion.* Telaprevir-based triple therapy was shown to be a long-term effective but complex treatment option for individual patients with hepatitis C graft. With the recent improvements in hepatitis C therapy options telaprevir may not be recommended as a standard therapy for this indication anymore.

## 1. Introduction

Hepatitis C Virus (HCV) recurrence after orthotopic liver transplantation (OLT) is the major cause of graft failure and death in HCV recipients [1]. Graft reinfection with accelerated fibrosis deposition occurs in all patients with detectable HCV ribonucleic acid (HCV RNA) at the time of transplantation, and 30% of these patients will develop graft cirrhosis within 5 years after OLT, with a mortality risk directly related to HCV recurrence of 15% [2, 3]. In addition to high HCV RNA levels in the early post-OLT period, factors such as HCV genotypes 1 and 4 as well as an older donor age, graft steatosis, the degree of human leukocyte antigen (HLA) matching, and the IL28B genotype of the donor and recipient have been identified as negative predictive factors for HCV recurrence [4, 5]. Obtaining a sustained virological response (SVR) by successful antiviral therapy can distinctly improve the graft and overall patient survival [6, 7]. Until 2011, the standard antiviral therapy regimen consisted of a dual therapy of pegylated interferon (PegIFN) and ribavirin (RBV). In addition to a poor tolerance, especially in transplanted patients, overall SVR was only achieved in one-third of HCV-positive recipients (−30% GT1, −50% GT5) [8, 9]. Therefore, establishing more efficient therapy regimens for patients with severe HCV recurrence remains essential. The introduction of the novel NS3/4 protease inhibitors (PI) boceprevir (BOC) and telaprevir (TVR) in 2011 had fundamentally changed the treatment options for HCV patients. Several clinical studies demonstrated that the addition of BOC or TVR to standard dual therapy in immunocompetent patients led to promising SVR rates even in patients who failed to achieve an SVR during previous treatment with PegIFN/RBV [10–12]. However, this first success of triple therapy was accompanied by a distinct increase of treatment-related serious adverse effects (SAE), such as manifest anemia, thrombocytopenia, neutropenia, bacterial infections, and decompensation of liver cirrhosis with a potentially life-threatening clinical course [13]. As an additional concern, PIs are metabolized via the cytochrome P450 (CYP) enzyme system and are substrates and inhibitors of the CYP 3A4/5 enzyme, as well as of the efflux pump P-glycoprotein (P-gp), and may therefore cause severe drug-drug interactions (DDIs) with a wide range of approved medications, including calcineurin inhibitors (CNIs) [14]. Therefore, the clinical application of PIs in the treatment of HCV recurrence after OLT is extremely challenging. Pharmacokinetic studies demonstrated that TVR exposure increased cyclosporine and tacrolimus levels 4.6- and 70-fold, respectively [15]. Thus, an intensified monitoring of CNI levels during TVR therapy is required to achieve a balance between toxicity due to insufficient CNI dose reduction and rejection due to disproportionate CNI dose reduction. Several studies have demonstrated the feasibility of combining PIs together with CNIs until today with reported SVR rates of 20% to 50% even in formerly considered hard-to-treat patients [16–19]. The recent rapid improvement in HCV therapy such as the introduction of new directly acting antivirals (DAA) and the possibility of IFN-free regimes has however led to a therapeutic hold in the clinical application of first generation PIs [20]. Due to these rapid developments in HCV treatment options clinical and academic research has of course also focused on the feasibility, management guidelines, and effectiveness of the novel anti-HCV agents. Reports of long-term results of first generation PI's treatment thus remain scarce. The aim of our study therefore was to report our results of the one-year follow-up after TVR/PegIFN/RBV triple therapy in combination with cyclosporine immunosuppression in GT1-infected relapsers and previous nonresponders after OLT.

## 2. Patients and Methods

### 2.1. Study Design

A retrospective analysis of 12 genotype 1 infected liver graft recipients with recurrent HCV who underwent TVR-based antiviral triple therapy between March 1, 2012, and July 31, 2013, at the Department of General, Visceral and Transplantation Surgery at Charité University Hospitals, Berlin, Campus Virchow, was performed. All patients included in this study had confirmed HCV recurrence with detectable HCV RNA in the PCR analysis and biopsy-proven graft fibrosis according to the Desmet and Scheuer classification (0, absent; 1, mild without septa; 2, moderate with few septa; 3, numerous septa without cirrhosis; and 4, cirrhosis) [21]. The indication to perform TVR-based antiviral therapy was based on sufficient renal function (glomerular filtration rate ≥ 60 mL/min according to the Cockcroft-Gault formula) and red blood cell count (hemoglobin levels ≥ 10 g/dL), as well as on adequate patient compliance. TVR treatment was not considered in patients with allograft cirrhosis, renal insufficiency, or general contraindications to IFN therapy. This study was performed in accordance with the Declaration of Helsinki and its amendments and approved by the institutional ethic committee. All patients were extensively educated about treatment-related side effects and the increased risk of DDIs during TVR therapy. Written consent to perform TVR/PegIFN/RBV triple therapy and consecutive PegIFN/RBV dual therapy by each patient was documented in the medical records.

### 2.2. Treatment Algorithm

The overall antiviral treatment duration was scheduled as 12 weeks of TVR/PegIFN/RBV triple therapy and 36 weeks of consecutive PegIFN/RBV dual therapy. Primary immunosuppression was switched from tacrolimus (TAC, Prograf®) to cyclosporine (CyA, Sandimmun Optoral®) prior to the beginning of treatment, and a sufficient therapeutic range for the CyA dosage was defined as a total body clearance (TBC) from 80 to 120 ng/mL depending on the time that had elapsed since OLT. In addition, all patients underwent a lead-in phase of 4 weeks of RBV (Copegus©; Roche) at a daily dose of 600 mg to estimate hematological and renal tolerance. Hemoglobin and serum creatinine levels were measured once a week during the lead-in phase, and the RBV dosage was eventually adjusted. Triple therapy was then begun with TVR (Incivo©; Janssen-Cilag International NV), PegIFN (Pegasys©; Roche), and RBV (Copegus©; Roche). TVR was given at either two daily doses of 1,125 mg or three daily doses of 750 mg, respectively, to reach a daily dose of 2,250 mg. The CyA dosage was reduced by 50% for each patient on the day that TVR treatment started.

### 2.3. Safety Assessment

Visits to our outpatient department for clinical examinations and laboratory measurements of CyA levels, blood count, and clinical chemistry renal and liver parameters were scheduled three times per week during the first two weeks and then two times a week until two weeks after the end of TVR treatment. The CyA dosage was adjusted to maintain a TBC of 80 to 120 ng/mL, and the RBV dosage was eventually reduced to 400 mg/day or even 200 mg/day, depending on the degree of cytopenia or renal function. Clinical and laboratory examinations were continued twice per month during PegIFN/RBV dual therapy. Patients were systematically screened for DDIs, as well as for treatment-related side effects. Erythropoietin (EPO) (Neorecormon©; Roche) and/or packed red blood cells (PRBCs) were administered in patients with hemoglobin levels below 6.21 mmol/L. Clinically manifest anemia and leukopenia were defined as hemoglobin levels below 4.97 mmol/L and leukocyte levels below 1.5/nL, respectively. In the event of SAEs of any type, antiviral therapy was discontinued immediately.

### 2.4. Treatment Efficacy and Definitions

To assess the treatment efficacy, HCV RNA was measured at baseline, at weeks 4, 8, 12, 24, and 48 of antiviral treatment, and then at weeks 12, 30, and 52 after the end of the treatment course (Roche Cobas AmpliPrep/Roche Cobas TaqMan, Roche Diagnostics GmbH, Mannheim, Germany; lower level of quantification (LLOQ) = 15 IU/mL). A rapid virological response (RVR) was defined as undetectable HCV RNA at week 4, and a complete RVR (cRVR) was defined as undetectable HCV RNA at weeks 4 and 12 of TVR-based antiviral therapy. An end of treatment response (ETR), an SVR 12, and SVR 52 were achieved when HCV RNA remained undetectable at the time of treatment discontinuation, 12 weeks or 52 weeks after the end of antiviral treatment, respectively. A Null Response (NR) was defined as a <2-log drop of HCV RNA at week 12, and a breakthrough (BT) was defined as the reappearance of HCV RNA at any time during treatment. A relapse was defined as undetectable HCV RNA at the end of treatment but detectable HCV RNA within 24 weeks of follow-up. Antiviral therapy was discontinued in the event of BT or if HCV RNA was not below 1000 IU/mL at week 4 of TVR therapy, in accordance with standard therapy guidelines.

## 3. Results

### 3.1. Patient Baseline Data

A total of 12 patients underwent TVR-based antiviral triple therapy between March 1, 2012, and July 31, 2013, at our institution. The mean patient age was 51.8 years (32–67 years), and the male-to-female ratio was seven (58%) to five (42%). All patients had undergone OLT due to HCV cirrhosis at our institution at a mean time of 63.7 months (13–190 months) prior to beginning TVR/PegIFN/RBV triple therapy. Ten patients had HCV genotype 1b, and two patients had genotype 1a. Seven patients had previously been treated with PegIFN/RBV. All patients had confirmed HCV RNA by PCR analysis and biopsy-proven HCV recurrence, with a fibrosis grade of 1 in four patients, a grade of 2 in five patients, and a grade of 3 in three patients. No patient had histological evidence of fibrosing cholestatic hepatitis or liver cirrhosis. Liver biopsies were performed within a median time of 252.5 days (105–927 days) prior to beginning TVR therapy. All patients had compensated liver and renal functions (Table 1).

### 3.2. Treatment Efficacy

**Figure 1:**
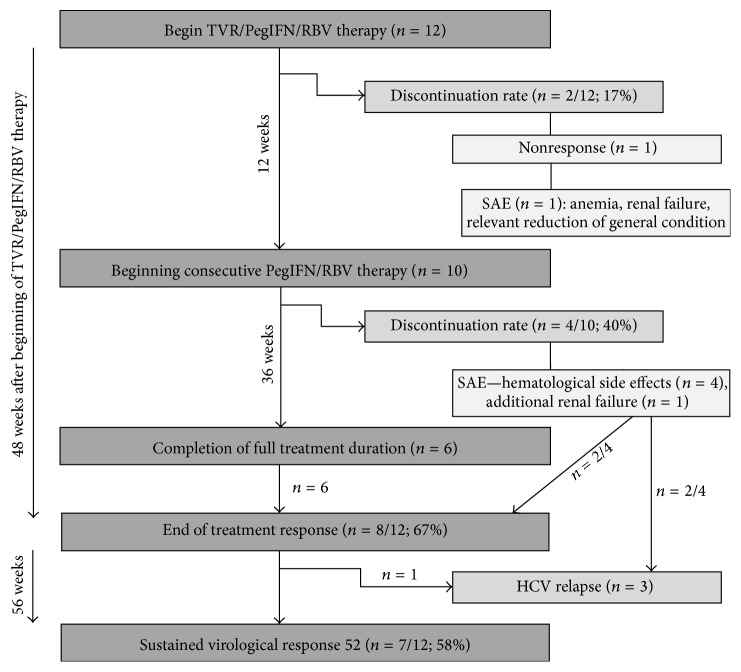
Treatment course and efficacy of 12 weeks of TVR/PegIFN/RBV triple therapy and 36 weeks of consecutive PegIFN/RBV dual therapy.

The mean HCV viral load at baseline was 6.1 log⁡10 IU/mL. Ten of the twelve patients (83%) completed the 12 weeks of TVR/PegIFN/RBV triple therapy, and six patients (50%) completed the intended overall treatment duration of an additional 36 weeks of consecutive PegIFN/RBV dual therapy. An RVR was achieved in 11 patients (92%), and all 10 patients (83%) who regularly finished TVR achieved cRVR. Forty-eight weeks after beginning TVR treatment, 8 out of 12 patients were HCV RNA negative (ETR 67%), including three patients who had prematurely discontinued the consecutive PegIFN/RBV dual therapy after 15, 26, and 26 weeks, respectively. TVR/PegIFN/RBV triple therapy was discontinued in one patient after 4 weeks due to a persisting HCV viral load of 2,700 IU/mL. Three patients had an HCV relapse, which was detected 36 days after the discontinuation of 8 weeks of TVR therapy in one patient and 16 and 29 days after discontinuation of 4 and 26 weeks of PegIFN/RBV dual therapy in two other patients. One patient experienced an HCV relapse 21 days after the regular end of 48 weeks of complete antiviral treatment. An SVR 12 and SVR 52 were then achieved in 7 out of the 12 patients (58%). All 7 patients had maintained the SVR in the two-year follow-up after the end of triple therapy (Figure 1).

### 3.3. Management of Immunosuppression Levels during TVR Therapy

**Figure 2:**
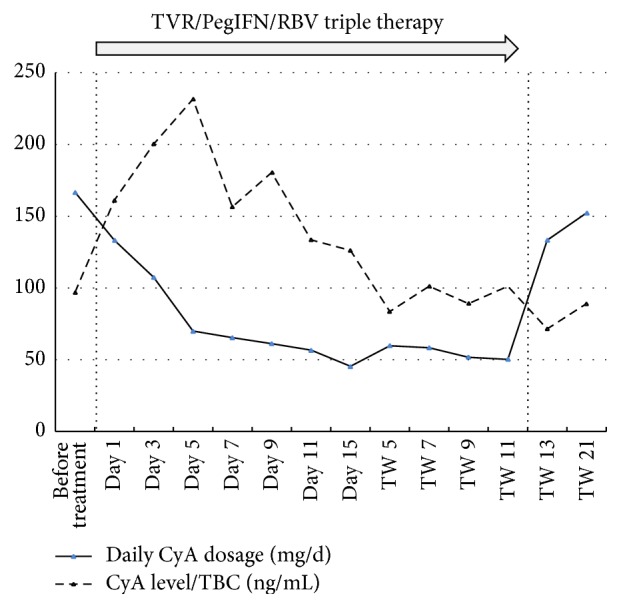
Course of immunosuppression dosage and levels during TVR/PegIFN/RBV therapy.

Eleven patients were switched from TAC to CyA-based immunosuppression within a mean time of 59.3 days (42–98 days) before beginning TVR therapy. One patient already had a primarily CyA-based immunosuppression. One week before beginning TVR therapy, the mean daily dose of CyA was 168.8 mg (100–250 mg), with a mean TBC of 96.9 ng/mL. The mean daily CyA dosage was reduced to 133.3 mg (50–250 mg; 75% of initial daily CyA dosage), 65.5 mg (50–150 mg; 41%), 45.4 mg (10–100 mg; 29%), and 50.3 mg (30–125 mg; 28%) after 1 day, 7 days, 14 days, and 12 weeks, respectively, of TVR treatment. Eight weeks after the end of TVR treatment, the mean daily CyA dosage was increased fourfold, to 152.3 mg (75–200 mg), compared with the ETR CyA dosage (Figure 2).

### 3.4. Treatment-Related Adverse Events

Out of the 11 patients who underwent TVR therapy for >4 weeks, 10 patients (91%) suffered from treatment-related adverse effects (AEs), and one patient had to discontinue TVR therapy after 8 weeks due to severe anemia, progressive renal decompensation, and relevant reduction of the patient's general condition. In the following antiviral treatment course, four patients had to discontinue PegIFN/RBV dual therapy because of severe declines in general patient conditions combined with manifest hematological side effects (anemia and leukopenia) in each of the 4 patients and an additional renal decompensation in one patient. Two of the 6 patients who completed the full treatment duration also required an RBV dose reduction to a daily dose of 400 mg or 200 mg, respectively. During the 12 weeks of TVR/PegIFN/RBV therapy, the mean hemoglobin level dropped from 7.4 mmol/L to 5.7 mmol/L, whereas the mean WBC count dropped from 4.8/nL to 3.4/nL, respectively. Five patients (45%) developed anemia with hemoglobin levels below 6.2 mmol/L and required EPO administration during this time period. Four of these patients also received blood transfusions. Granulocyte colony-stimulating factor (GC-SF) was given to 5 patients due to a WBC below 2.5/nL. We did not observe a decrease of GFR under TVR therapy. The median GFR decreased from 72.0 mL/min/1.73 m^2^ to 58.6 mL/min/1.73 m^2^ from the beginning to the end of the TVR/PegIFN/RBV triple therapy. In addition, 3 patients (27%) developed infections: urinary tract infections in 2 patients and clostridium difficile diarrhea in 1 patient (9%). Skin changes expressed by a mild rash were observed in 2 patients (18%), and 1 patient (9%) reported a disturbing anorectal pruritus (Table 2).

## 4. Discussion

The introduction of PIs has fundamentally changed the treatment options for patients with primary HCV and HCV recurrence after OLT even in GT1 patients with a prior nonresponse to antiviral therapy and may thus be considered as a first step towards modern HCV therapy [10, 22]. The promising results regarding the antiviral efficacy of this rather revolutionary treatment approach have, however, been overshadowed by an increase in directly treatment-associated AEs, especially in immunocompromised patients, in whom dual therapy has already proven particularly challenging [8, 23–25]. At the time our study began, the two PIs TVR and BOC were approved for antiviral therapy in Europe. The therapy costs were similar, but as previous authors have noted, TVR appeared to be more “streamlined” to us in comparison to BOC [26]. In addition, Benito et al. also reported that triple therapy with TVR exhibits greater early antiviral activity than that with BOC which has been demonstrated to be a principal predictive factor for consecutive SVR [27, 28]. Morisco et al. recently also reported that RVR was the only independent predictive factor of antiviral response in cirrhotic patients treated by triple therapy with TVR. An even shorter therapy may thus be considered [29].

In our study, we analyzed the antiviral efficacy and safety of TVR/PegIFN/RBV triple therapy in HCV GT1-infected liver transplant recipients, a patient cohort in whom SVR rates after former standard dual therapy were rather low in comparison with genotypes 2 and 3. In our study, we showed that despite the complexity in this setting, TVR-based triple therapy was feasible and efficient also in the long-term outcome. Eight of the 12 patients (67%) achieved an ETR, including 2 patients who had to discontinue consecutive dual therapy prematurely due to treatment-related side effects. One patient had an HCV relapse 21 days after regularly finishing the 48-week complete antiviral treatment course. An SVR 12 and SVR 52 were then obtained in 7 of these patients (56%). Our results are in accordance with Faisal et al., who analyzed TVR-based triple therapy for HCV recurrence after OLT in a multicenter trial and reported an ETR of 75% and an SVR 12 of 61.5% [19]. As an important and novel finding of our study in comparison to other reported results all patients with an SVR 12 also achieved an SVR 52. Severe PI's treatment-related AEs including death have been reported in several studies [19, 24, 30, 31]. In our study, 10 of the 11 patients who underwent TVR therapy >4 weeks suffered from treatment-related AEs. These AEs were clinically severe enough to discontinue TVR therapy in one patient and consecutive dual therapy in 4 additional patients. The treatment discontinuation rate in our study is comparable to the results of Gallegos-Orozco in nontransplanted (high-risk) cirrhotic patients (24%), thereby underlining the particularly difficult conditions for TVR therapy in immunocompromised patients [32]. In our study, hematological side effects were the most common treatment-related AEs. Five patients developed anemia, all of whom required EPO administration and 4 of whom required blood transfusions. Triple therapy is associated with a 20% increase in the incidence of treatment-related anemia compared with the former standard PegIFN/RBV therapy, and, according to a recent interim analysis of patients who underwent TVR therapy after OLT, 77% of all patients require EPO administration under TVR therapy [33–35]. Additionally, 5 patients in our cohort had leukopenia and required G-CSF administration. Both anemia and leukopenia are most likely explained by the bone marrow-suppressive and nonimmune hemolytic anemia effect of RBV, which is aggravated when RBV is given in combination with TVR [24, 36]. In our study, two patients also had to discontinue antiviral therapy due to severe renal decompensation, which may also be explained in the context of RBV therapy [37]. The RBV dosage may be adapted to hemoglobin and GFR levels under PI therapy with no negative effect on later SVR rates [38]. A complete cessation of RBV, however, requires a discontinuation of TVR therapy and therefore needs to be carefully evaluated. In our study, RBV dose reduction was necessary in two of the six patients who completed the full antiviral treatment duration; one of these patients experienced HCV relapse 21 days after the regular end of the 48-week antiviral treatment course. In an attempt to assess hematological and renal tolerance before beginning TVR therapy, all patients in our study underwent a 4-week RBV lead-in phase. It appears to be reasonable to include IFN in this lead-in phase, not only to assess general therapy tolerance but also to possibly identify interferon-insensitive patients who are at risk of developing protease-resistant HCV strains during TVR therapy [39]. Patients at high risk for SAEs or virological nonresponse may thereby be identified before beginning expensive and potentially harmful TVR therapy. Another major issue for TVR therapy in transplanted patients is the complex management of immunosuppression. Dose reductions and adjustments of CNIs are required during the entire TVR treatment course due to severe DDIs. In addition to the risk of CNI toxicity, previous studies have also reported rejection episodes in 4 to 6% of patients during antiviral therapy [40, 41]. Generally, the CyA dosage appears to be less difficult to manage because of the slighter required dose reduction compared with TAC [23]. Additionally, an additional antiviral action of CyA has been described due to the involvement of cyclophilin A in the viral replication of HCV, which is underlined by the results of a meta-analysis showing that during dual therapy more patients achieved SVR with CyA than with TAC [42, 43]. All of our patients were therefore switched to CyA before beginning TVR therapy. Despite deviations in the total CyA body clearance (TBC), especially in the first days of TVR therapy, no patient developed signs of toxicity or rejection. Of course, our study is limited by the small sample size and the lack of randomization. Additionally, our patients were all well selected with regard to their general conditions and a rather less distinct fibrosis progression in comparison with studies that included rather difficult-to-treat patient populations, including patients with cirrhosis or cholestatic hepatitis [24, 44]. However, we may still conclude that TVR therapy in general as well as the concomitant management of immunosuppression may safely be performed in HCV GT1-infected liver graft recipients with promising SVR 52 rates. In the context of the rapid developments in HCV treatment options in the last years, the clinical relevance of first generation PIs has however to be considered as rather negligible nowadays. The recent introduction of novel next-generation antiviral agents such as sofosbuvir, daclatasvir, simeprevir, ombitasvir, paritaprevir, and dasabuvir has led to a fundamental change in the treatment perspectives of modern HCV therapy [45, 46]. Several clinical studies have demonstrated that a combination of these new drug classes now allow interferon-free treatment regimens with a less harmful SAE spectrum, a superior antiviral efficacy, and no significant interference with immunosuppression [47–50]. The EASL guidelines for HCV treatment before and after liver transplantation have therefore recently been adapted [51]. However, the worldwide replacement of PIs by novel DAAs is until today amongst other factors limited by economic burdens. Long-term results of PI therapy efficacy as well as the emergence of resistance thus need to be continuously analyzed in order to allow an optimal and evidence-based HCV treatment also in health care systems with limited access to recent achievements of modern HCV therapy.

## Figures and Tables

**Table 1:** Patient characteristics at baseline.

	Included patient population for TVR/PegIFN/RBV triple therapy (*n* = 12)
Age (years), mean ± SD	51.8 ± 10.5
Gender (male)	7 (58%)
Body mass index (kg/m^2^), mean ± SD	26.4 ± 5.3
HCV genotype	
1a	2 (17%)
1b	10 (83%)
Previous PegIFN/RBV therapy after OLT	
Naive	5 (42%)
Nonresponder/relapsers	7 (58%)
Time between OLT and beginning TVR/PegIFN/RBV therapy (months), mean ± SD	63.7 ± 61.4
Fibrosis grade	
1	4 (33%)
2	5 (42%)
3	3 (25%)
HCV viral load (log 10 IU/mL), mean ± SD	6.1 ± 0.8
Bilirubin (*μ*mol/L), mean ± SD	23.9 ± 13.7
ALT (*μ*kat/L), mean ± SD	0.99 ± 0.84
Glomerular filtration rate (mL/min/1.73 m^2^), mean ± SD	72.0 ± 20.4
Hemoglobin (mmol/L), mean ± SD	7.33 ± 1.43
White blood cell count (/nL), mean ± SD	4.8 ± 1.9
Platelet count (/nL), mean ± SD	222.3 ± 105.9

**Table 2:** Treatment-related adverse events during TVR/PegIFN/RBV triple therapy.

	TVR/PegIFN/RBV triple therapy > 4 weeks (*n* = 11)^*∗*^
Overall treatment-related AEs during TVR/PegIFN/RBV	10 (92%)
Discontinuation of TVR/PegIFN/RBV due to AEs	1 (9%)
Anemia with hemoglobin levels below 10 g/dL	5 (45%)
EPO administration	5 (45%)
Blood transfusion	4 (36%)
Leukopenia with a WBC count below 1.5/nL	5 (45%)
GCF administration	5 (45%)
Renal failure	2 (18%)
Infection	3 (27%)
Skin changes	2 (18%)
Anorectal pruritus	1 (9%)
Death	0

^*∗*^TVR/PegIFN/RBV triple therapy was discontinued in 1 patient after 4 weeks due to a nonresponse.
